# Cervical Cancer Protection in Japan: Where Are We?

**DOI:** 10.3390/vaccines9111263

**Published:** 2021-11-01

**Authors:** Asami Yagi, Yutaka Ueda, Mamoru Kakuda, Satoshi Nakagawa, Kosuke Hiramatsu, Ai Miyoshi, Eiji Kobayashi, Toshihiro Kimura, Megumi Kurosawa, Manako Yamaguchi, Sosuke Adachi, Risa Kudo, Masayuki Sekine, Yukio Suzuki, Akiko Sukegawa, Sayaka Ikeda, Etsuko Miyagi, Takayuki Enomoto, Tadashi Kimura

**Affiliations:** 1 Department of Obstetrics and Gynecology, Osaka University Graduate School of Medicine, Osaka 565-0871, Japan; a.yagi@gyne.med.osaka-u.ac.jp (A.Y.); mamorukakuda@gyne.med.osaka-u.ac.jp (M.K.); s.nakagawa@gyne.med.osaka-u.ac.jp (S.N.); hiramatsu@gyne.med.osaka-u.ac.jp (K.H.); aimiyoshi20090808@gyne.med.osaka-u.ac.jp (A.M.); ekobayashi@gyne.med.osaka-u.ac.jp (E.K.); binbin@gyne.med.osaka-u.ac.jp (T.K.); tadashi@gyne.med.osaka-u.ac.jp (T.K.); 2 Department of Obstetrics and Gynecology, Niigata University Graduate School of Medical and Dental Sciences, Niigata 951-8510, Japan; m-kurosawa@med.niigata-u.ac.jp (M.K.); manako0131@gmail.com (M.Y.); sadachi@med.niigata-u.ac.jp (S.A.); pearpear@med.niigata-u.ac.jp (R.K.); masa@med.niigata-u.ac.jp (M.S.); enomoto@med.niigata-u.ac.jp (T.E.); 3 Department of Obstetrics and Gynecology, Yokohama City University School of Medical, Yokohama 236-0004, Japan; yetii@yokohama-cu.ac.jp (Y.S.); aki69kin@yokohama-cu.ac.jp (A.S.); emiyagi@yokohama-cu.ac.jp (E.M.); 4 Division of Cancer Statistics Integration, Center for Cancer Control and Information Services, National Cancer Center, Tokyo 104-0045, Japan; sayakaikeda0201@gmail.com

**Keywords:** HPV vaccine, cervical cancer, government suspension of recommendation, adverse events, vaccine hesitancy

## Abstract

In Japan, government subsidies for human papillomavirus (HPV) vaccination of girls aged 13–16 commenced in 2010. By early 2013, vaccination had become a widely accepted national immunization program. However, in June of 2013, the Ministry of Health, Labor, and Welfare (MHLW), the government’s lead agency, suspended its recommendation for vaccination in response to reports of adverse vaccine events. The rate of HPV vaccination quickly dropped from 70% to almost zero, where it has lingered for eight years. In 2020, a new 9-valent HPV vaccine was licensed in Japan. The momentum seemed to be building for the resumption of HPV vaccinations, yet Japanese mothers remain widely hesitant about vaccinating their daughters, despite the well-proven safety and efficacy of the HPV vaccines. The Japanese government and our educational and medical institutions must work harder as a team to inform our parents and their children about the life-saving benefits of the HPV vaccine, and at the same time, we must respond to all their concerns and questions. The vaccine hesitancy of unvaccinated women born in 2000 and thereafter is a natural consequence of the suspension of the government‘s recommendation. We must also take every possible measure to reduce the significant risk for cervical cancer these women have.

## 1. Introduction

Cervical cancer is one of the most common cancers for women. In 2020, an estimated 600,000 women were diagnosed with cervical cancer worldwide, and about 341,000 deaths were attributed to it [1]. On a global scale, Japan has been classified as having a moderate age-standardized incidence rate of cervical cancer; India, Nigeria, and other countries belong to this moderate group as well. Interestingly, China, Korea, Russia, and Brazil have a lower incidence rate. The World Health Organization (WHO) has noted that the burden of cervical cancer typically falls most heavily on women who lack proper access to health services, mainly those in low-and middle-income countries [2], thus, it is disconcerting that among the most developed countries of the world, Japan’s incidence rate of cervical cancer has been accelerating since 2000 (Age-adjusted rate: 28.0 in 1976, 9.1 in 2000, 14.1 in 2012, Annual percent change in 2000–2012: 3.8 (95% CI: 2.7–4.8)) [3], a trend not seen in any other advanced country. In Japan, 10,978 women were diagnosed with cervical cancer in 2018; in 2019, 2921 died from it [4,5]. Modern changes in sexual lifestyles and an increasing rate of smoking among women have clearly contributed to this national trend for increasing cervical cancer morbidity and mortality. Because Japan’s human papillomavirus vaccination (HPV) vaccination program was only started in 2010, the bulk of these cancers are occurring in an extended generation of unvaccinated women.

It is well established that the most critical risk factor for cervical cancer is having experienced a persistent infection with one of the high-cancer-risk versions of HPV [6]. Persistent infection can lead to developing precancerous lesions that, if undetected and untreated, can progress to invasive cervical cancer. HPV has retrospectively been detected in most cervical cancers in Japan [7,8]. The most frequent types attributed to causing cervical cancers are the HPV 16 and 18 strains, which together account for almost 60% of cervical cancers in Japan, which is significantly lower than the global average of 71% for HPV 16/18 [9,10]. Heavy smoking, long-term use of oral contraceptives, and promiscuous sexual experiences at a young age are contributing risk factors [11,12,13].

Most cervical cancers are primarily preventable by an effective HPV vaccination. Secondary prevention approaches include early and consistent screening for and treatment of precancerous lesions. Almost inexplicably, although Japan has in hand all the technical, medical, policy, and fiscal tools with which to eliminate cervical cancer, they are almost totally failing to do so.

Among its many problems is cervical cancer screening, which in Japan is recommended for all women over the age of 20. Incredibly, the screening rates for young women within three key age groups, of 20–24, 25–29, and 30–39, are only 10%, 10–20%, and 10–30%, respectively [14]. Forty-two-point-four percent of Japanese women aged 20–69 had a Pap smear in the past two years, while 60.7% of target women had Pap smear in the past three years on average across OECD countries [15]. Furthermore, Japan’s once enviable 70% HPV vaccination rate has fallen to almost zero [16], making Japan an outlier among the developed nations.

## 2. Politics, Policies, and Events Related to HPV Vaccinations

We would like to share some relevant background information, some insights on the current states of HPV vaccinations and cervical screening in Japan, and give some consideration as to what directions Japan is now moving. We begin by noting that the quadra-valent HPV vaccine was approved in 2006 in the United States of America, the European Union, and Australia, and by 2020, it had been approved in more than 130 countries [17]. The new broader-spectrum 9-valent vaccine has already been approved in more than 80 countries [18]. In 2018, the Director-General of WHO called for a global action to eliminate cervical cancer. In 2020, the World Health Assembly adopted its Global Strategy to accelerate the elimination of cervical cancer as a public health problem, which has since been adopted by many countries [2].

Table 1 shows a brief history of HPV vaccination in Japan. Here are some of the high points. In October of 2009, the bivalent HPV vaccine was the first to be cautiously licensed in Japan; licensing the quadra-valent form followed two years later, in July 2011, a full five years after it had been widely accepted elsewhere. Subsidies from local and national government programs for HPV vaccination of girls aged 13–16 commenced flowing in November of 2010. By April of 2013, both the bi-valent and quadra-valent HPV vaccines were being used routinely for vaccinating girls aged 12–16 as part of the National Immunization Program.

Those were the ‘good old days’ when vaccination rates for girls of targeted age groups approached 70% compliance. However, soon thereafter, the news media began to sensationalize reports regarding the occurrence of diverse symptoms after HPV vaccination, including chronic pain, motor impairment, and other symptoms. As a result, on the 14th of June 2013, Japan’s Ministry of Health, Labor, and Welfare (MHLW) decided to temporarily suspend the national immunization program’s recommendation for routine HPV vaccination while an investigation into the safety of the vaccine was conducted.

Such a safety study was carried out. In 2014, MHLW’s advisory group, the Vaccine Adverse Reactions Review Committee (VARRC), assessed the pathogenesis and the causality of the “diverse symptoms” reported to have occurred after HPV vaccination, and they defined them as “functional somatic symptoms”. Later that same year, the Japanese Pediatric Society submitted a request to the MHLW to resume its recommendation for HPV vaccinations, with no success. In 2015, positive results from an adverse vaccine events follow-up survey were released, but the suspension remained under “continued review”. Next, the WHO Global Advisory Committee for Vaccine Safety (GACVS) noted that the MHLW’s policy decisions were based on weak circumstantial evidence, causing the public to fail to use a safe and effective vaccine, potentially resulting in real harm [19]. In April 2016, a group of 17 academic societies, comprising the Japanese Expert Council on Promotion of Vaccination, published its “Views of Relevant Academic Groups on Promoting HPV Vaccination”, which stated that “the suspension of the governmental recommendation is an extremely alarming situation, and the Japanese government should resume its proactive support for HPV vaccination” [20].

A year later, in April 2017, a nationwide epidemiological survey, led by a research team designated by the MHLW, concluded that “the diverse symptoms” occurred equally among unvaccinated girls of the same age groups and were therefore not specific to vaccinated girls [21]. The details of their findings are described in the next section.

In response to this last finding, in 2017 the VARRC discussed how to provide better information to the public to overcome their deeply entrenched HPV vaccine hesitancy. The following year, 2018, a HPV vaccine information leaflet developed by the MHLW was released; an evaluation of its results was reported in 2019 [22,23].

Most recently, in 2020, a 9-valent HPV vaccine was licensed in Japan. Combined with the pro-vaccine effort driving the COVID-19 vaccination program, momentum seems to be building for the resumption of HPV vaccinations as well. However, eight long years have now passed since the recommendation was suspended, and during those eight years, almost no one was vaccinated, leaving large numbers of Japanese girls and women at future risk of HPV-driven cancers and cancer deaths.

## 3. Safety and Efficacy of HPV Vaccination

In 2013, news of Japan’s suspension of its HPV vaccine recommendation due to its concerns about vaccine side-effects traveled globally through online news and social media networks [24]. As a result, some countries saw a decline in vaccination rates, but no other country stopped recommending it, as Japan had done. In 2015, HPV vaccine safety reports were released, one after another. The US Centers for Disease Control and Prevention stated that the HPV vaccine was proven to be safe [25]. The European Medicines Agency stated that there was no evidence that the HPV vaccine had caused Postural Orthostatic Tachycardia Syndrome or complex regional pain syndrome [26]. The French National Agency for Medicines and Health Products Safety reported that increases in the risk for Guillain-Barré Syndrome were limited and that the risk for 14 other autoimmune diseases had not increased overall [27]. In 2018, after careful review, Cochrane reported that HPV vaccination was not found to have increased the risk of serious adverse reactions in women aged 15–26 [28].

In Japan, two studies on the safety of HPV vaccines were conducted. The first was a nationwide epidemiological survey by a research team designated by the MHLW, the results which were reported to the VARRC in December 2016. Since the present Japanese vaccination system does not follow a systematic process for identifying adverse reaction causality, a questionnaire survey was conducted among participating medical institutions. That study had certain limitations: the temporal relationship between the vaccination and the symptom onset was unclear in many cases, and the age bias by vaccination history was too large to allow for age adjustment. In addition, because it is a questionnaire survey, it was suggested that the study held various biases, such as the content of the medical interviews. Although the study design did not allow for an in-depth analysis of symptom causality, it concluded that unvaccinated girls of the same age were getting the same “diverse symptoms” [21,22].

The second safety study was the Nagoya study, conducted in 2016 to analyze whether there was an association between HPV vaccination and the development of 24 reported post-vaccine symptoms included: irregular menstrual periods; an abnormal amount of menstrual bleeding; pain in the joints or other parts of the body; severe headache; fatigue; poor endurance; difficulty concentrating; visual field abnormalities; abnormal sensitivity to light; sudden vision loss; dizziness; cold feet; difficulty falling asleep; abnormally long sleep duration; skin problems; hyperventilation; memory decline; loss of ability to perform simple calculations; loss of ability to remember fundamental Kanji [Chinese characters indispensable for life in Japan]; involuntary uncontrollable body movements; loss of the ability to walk normally; becoming dependent on a walking stick or wheelchair; sudden loss of strength; and weakness in the hands and feet [29]. The survey was conducted by Nagoya City in response to a request from the Aichi Branch of All Japan Coordinating Association of HPV Vaccine Sufferers. The questionnaire included items that were selected after consultations with both the study investigators and the requesting body. In total, 29,846 women born between 2 April 1994, and 1 April 2001, responded. No significant increases in the occurrences of any of the twenty-four post-HPV vaccine symptoms of interest were found. The results of the Nagoya vaccine-safety study were the strongest evidence to date that there was no causal association between HPV vaccination and any of the twenty-four most commonly reported post-vaccine symptoms.

The next question is about the efficacy of the HPV vaccines, of which there have been many reports. Two studies, in particular, are noteworthy in that they explored the efficacy of the HPV vaccine in preventing the most invasive and deadly forms of cervical cancer. In 2018, Luostarinen et al. were the first to report on evidence obtained from an intention-to-treat trial showing that HPV vaccination strongly protects against HPV-associated invasive cervical cancer [30]. They analyzed data from the Finnish Cancer Registry that used 7-year follow-up time periods between June 2007 and December 2015. Ten cases of HPV-associated invasive carcinomas occurred in 17,838 non-HPV vaccinated women (124,245 women-years). There were no cases of invasive carcinomas in 9529 HPV vaccinated women (65,656 women-years).

In 2020, Lei, et al., in another large-scale follow-up study, also investigated the efficacy of the HPV vaccine for the prevention of invasive cervical cancer [31]. They used data from 2006 through 2017 from Swedish demographic and health registries to follow an open population of 1,672,983 girls and women who were from 10 to 30 years of age during their study period. The analysis was controlled for age-at-follow-up, calendar year, county of residence, and parental characteristics. After adjustment for all these covariates, the incidence rate ratio for the comparison of the vaccinated population with the unvaccinated population was 0.12 (95% CI, 0.00 to 0.34) among women who had been vaccinated before the age of 17 years. In the future, more countries that have successfully introduced an HPV vaccination program will report on its effectiveness in preventing invasive cervical cancer.

In Japan, the preventive effect of the vaccine for stages of cervical cancer up to stage CIN3 has been reported. HPV vaccination led to a significant reduction in the incidence of abnormal cytology in vaccinated women born in 1994–1995 [32]. In a case-control study the size of one-tenth of the total Japanese population, a preventive effect of CIN2 was reported [33]. In research conducted in Matsuyama City, a preventive effect of CIN3 was reported [34]. In a generation of unvaccinated women, the incidence of CIN3 or higher was 0.09% (7/7872), compared to a later cohort of women with a vaccination rate of 79.0%, where no CIN3 or worse cases were found (*p* = 0.016).

An expanded survey showed similar results. Data was collected for women born from 1990 to 1997 who became eligible for their 20-year-old cervical cancer screening between 2010 to 2017. The incidence of CIN3+ that occurred in the generation before the HPV vaccine was introduced into Japan was 0.17% (29/17,040); for the generation that was vaccinated, the CIN3+ rate was 0.02% (2/8155), indicating significant protection (*p* = 0.0008) was afforded by the vaccine [35].

These studies have clearly established the safety and efficacy of the HPV vaccines. Thus, a strong case can be made, and without further hesitation, re-implementing Japan’s national HPV vaccination program.

## 4. Future Risk Caused by the Governmental Recommendation for HPV Vaccination

There is a clear need for maintaining vaccine-safety vigilance, and this is not the first time Japan has temporarily suspended the use of a vaccine. In 1975, two highly publicized cases of death after diphtheria, pertussis, and tetanus (DPT) vaccination triggered the suspension of DPT vaccination. It was resumed three months later, but the vaccination rate dramatically decreased. In 1979, 13,000 cases of DPT and more than 20 DPT-related deaths were reported [36]. Similar problems occurred with the Japanese measles, mumps, and rubella, encephalitis, and haemophilus influenzae type b and pneumococcal vaccines. Each incident has affected the overall Japanese vaccination system and generated accompanying vaccination gaps.

Vaccine hesitancy was listed as the eighth of the “ten greatest threats to global health” that WHO announced in 2019 [37]. In Japan, confidence in the HPV vaccine was completely destroyed in 2013. For girls born in or after 2000, who therefore reached the age for free HPV vaccination only after the suspension of the governmental recommendation for it, their HPV vaccination rates have been in dismal decline, from the high of almost 70% achieved in 2013, despite the vaccination continuing to be free throughout free. The cumulative vaccination rates for girls born between 2000 and 2003 were 14.3%, 1.6%, 0.4% and 0.2%, respectively [16].

**Figure 1:**
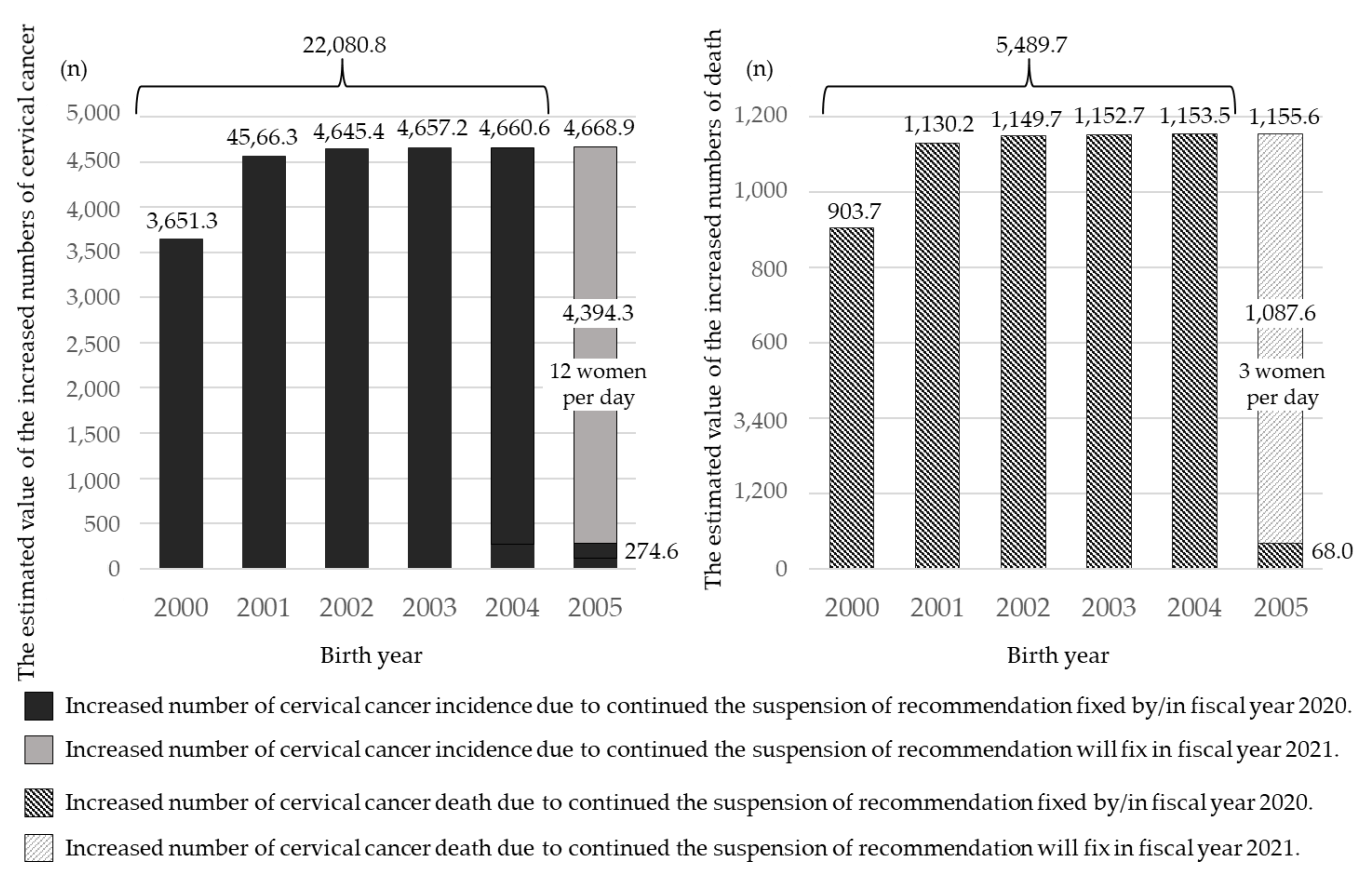
The estimated value of the increased numbers of incidence and death.

Based on several assumptions, we have calculated the potential for future cervical cancer incidence and mortality numbers that will be increased as a result of the policy decision to continue not recommending HPV vaccination [38] (Figure 1). The increased numbers of future incidences of cervical cancer, in each birth year, compared to those of females born in 1999, most of whom received HPV vaccination with public subsidies before the suspension of the recommendation, were estimated as follows: 2000: 3651.3, 2001: 4566.3, 2002: 4645.4, 2003: 4657.2, 2004: 4660.6, 2005: 4668.9. The increased numbers of future deaths of cervical cancer were also estimated, as follows: 2000: 903.7, 2001: 1,103.2, 2002: 1149.7, 2003: 1152.7, 2004: 1153.5, 2005: 1155.6. If the situation does not improve in 2021, the potentially increased total number of cervical cancers was estimated to be 22,080.8, with about 5489.7 deaths. Simms et al. have done a similar analysis and evaluated the expected number of cervical cancer cases and deaths over the lifetime of cohorts born from 1994 to 2007 [39]. The vaccine gap that occurred from 2013 to 2019 was predicted to result in an additional 24,600–27,300 cases of cervical cancer and 5000–5700 deaths over the lifetime of the cohort of women born between 1994 and 2007, compared with such cases if vaccine coverage had remained at 70% since 2013.

It is highly likely that these estimates of future disease will become a reality if sufficient measures are not taken to protect those women who missed their opportunity to be vaccinated due to the suspension of governmental recommendations. A significant rise in abnormal cervical cytology findings in the post-2013 cohort of unvaccinated women will demonstrate the effectiveness of the HV vaccine—in a very undesirable way. If this happens, there is deepening concern that the number of cervical cancer cases and deaths will increase in parallel in the future—all due to the decline in vaccination rates that has occurred under the suspension of the HPV vaccine recommendation.

## 5. Future Prospects for Discussion

### 5.1. After a Resumption of the Governmental Recommendation?

Eight years have passed since the government’s recommendation was discontinued in 2013. During this time, the population of mothers with daughters eligible for HPV vaccination has been almost completely replaced. What are now the attitudes toward HPV vaccination of this new generation of mothers?

Following the suspension of governmental recommendation, we have been periodically conducting internet surveys of the mothers, which were done in March 2014, May 2015, March 2016, and December 2019, and compared the results of these surveys [40]. The respondents were mothers who had HPV-unvaccinated daughters who were aged 12–16 at the time of their responses. The results showed that at least 90% or more of the mothers knew something about the HPV vaccine throughout the four surveys: 95.0% (190/200), 97.5% (2008/2060), 89.8% (1796/2000), and 91.7% (1416/1545), respectively. Similarly, the percentage of mothers who knew about the 2013 news reports regarding so-called adverse events remained high over the course of the four surveys: 83.0% (166/200), 80.9% (1666/2060), 78.7% (1573/2000) and 85.9% (1328/1545), respectively. The percentages of mothers who replied they would “inoculate” if the MHLW restarted their recommendation was 22.5% (45/200), 21.0% (433/2060), 12.2% (244/2000), and 17.8% (275/1545). The percentages were significantly higher during the 1st and 2nd surveys and became significantly lower for the 3rd survey (*p* < 0.05, *p* < 0. 05, *p* < 0.05, respectively). At any point in time, only about 20% of mothers would “inoculate” solely based on whether the MHLW would restart their recommendation.

Most Japanese mothers are concerned about vaccinating their daughters against HPV, so there is still hope for a turnaround. We must plan now to quickly take effective measures to increase the vaccination rate, once the long-promised governmental recommendation is resumed. As part of that preparation, we have been investigating the effectiveness of providing different informational leaflets to girls of HPV vaccination age and/or to their parents [41]. In July 2019, Isumi City (population 38,000) started sending an informational/educational leaflet addressing the risks of cervical cancer. Their leaflet was sent to 139 girls whose birth year was 2003 and who were 16 at the time. Their cumulative vaccination rate reached 10.1% (14/139) by December 2019, which was significantly higher than the 0.0% for girls born in 2002 who did not receive the leaflet (*p* < 0.001). This was proof that an increase in HPV vaccination rate could be achieved (among the targeted girls born in 2003) by a leaflet sent to individuals by a local government entity.

We have found that, even under the suspension of governmental recommendation, if their family doctor provided information using an information leaflet explaining the need for cervical cancer prevention, that act led to an increase in the number of vaccinations [42]. Among the 53 mothers who said they would impose certain preconditions before being willing to encourage their daughters’ HPV vaccination, 21 (39.6%) became more willing to vaccinate their daughters immediately after receiving such an explanation. Mothers requiring no other preconditions, other than the resumption of the governmental recommendation, were more likely to be willing to be vaccinated after receiving their doctor’s explanation (*p* = 0.02). Seven of the 21 mothers (33.3%) returned to the clinic to get their daughter vaccinated during the study period. Despite the small number of subjects observed, the study suggests that efforts to re-establish HPV vaccine acceptance should be focusing on mothers who are less likely to impose preconditions on their daughter’s vaccination.

The challenges Japan faces as a nation in reviving its national HPV vaccination program are immense. How can we possibly achieve the WHO’s lofty goal of 90% HPV vaccination by age 15 [2]? According to one marketing theory, when the penetration (acceptance) rate of a product or service reaches 16.0% and overcomes the next chasm of penetration, demand will accelerate rapidly. This marketing theory is useful in considering the re-promotion of the HPV vaccine [43]. Under this theory, the adopter categories by which a new product penetrates the market can be divided into five groups, including Innovators, Early adopters, Early majority, Late majority, and Laggards.

According to the “Chasm Theory”, there is a large chasm between the early market, consisting of Innovators and Early adopters, and the mainstream market, which consists of the Early and Late majorities, that cannot be easily crossed. The leaflet intervention by Isumi City resulted in a 10% vaccination rate, and the vaccination rate, if and when the recommendation is resumed, may be about the same. As the report by Ugumori et al. implies, to cross that chasm, first, medical workers will need a firmer understanding of the HPV vaccine so that they can confidently recommend vaccination to the targeted population of girls and their parents. That population will be expected to pass on that recommendation to their peers, resulting in the widening dissemination of the HPV vaccine among the majority.

### 5.2. Women Who Missed Their Opportunity of HPV Vaccination

If the governmental recommendation is finally resumed in 2022, it will be the women born between 2000 and 2005 that have a very low vaccination rate because they have surpassed the targeted age (12–16) for the routine free vaccination program. When the future risk of cervical cancer incidence in women born in 1993 is considered to be 1 (these girls were never vaccinated because they were over 16), for receiving HPV vaccination when public subsidies started in 2010, the risk for the women born in 2000 is 0.916, 0.990 for women born in 2001, 0.998 for women born in 2002 to 2003, and 0.999 for women born in 2004 to 2005 [43]. These women are between the ages of 17 and 22, the age range where sexual experiences increase. The cumulative rate of first intercourse among Japanese women is 30% at age 18, over 50% at age 19, and reaches 70% at age 22 [44].

The introduction of catch-up vaccinations has been somewhat effective, although it can never be as effective as the original preventive effect due to the increasing rate of sexual experience. In Sweden, the incidence rate ratio for the comparison of the vaccinated population with the maximally unvaccinated population was significantly reduced to 0.47 (95% CI, 0.27 to 0.75) among women who had been vaccinated between the ages of 17 to 30 [31]. In order to reduce their risk, it would be desirable for them to receive the 9-valent vaccine, free of charge and as soon as possible, although it has not yet been introduced into the routine vaccination system.

Women who lost their opportunity to be vaccinated also lost the opportunity to know how to prevent cervical cancer. We analyzed two internet surveys, conducted in 2015 and 2020, to evaluate the differences in their understanding of the means of preventing cervical cancer in young Japanese women [45]. We compared non-vaccinated women aged 18–19 years during the golden period of pro-vaccination policies and the period when the recommendation was suspended. Even though none of the women in either group were HPV-vaccinated, 98.8% of the pro-vaccination generation knew about the HPV vaccine, while only 60.3% of the women under the suspension of recommendation were aware of the vaccine (*p* < 0.001). The answer yes to the question “Did you ever talk with your family about cervical cancer?” was also significantly lower (25.0%) compared to 44.6% of the pro-vaccination generation (*p* < 0.001).

For those who missed their routine vaccination opportunity, we must promote providing them with catch-up vaccination opportunities, and we must encourage them to undergo routine cervical cancer screening. To avoid burdening them unfairly with the consequences of the suspension of the government recommendation, every measure must be taken to reduce their risk.

## 6. The Real Harm Caused by Vaccine Hesitancy

Following the results of our risk predictions in 2016, we have strongly and persistently been appealing for a resumption of the governmental recommendation, but it has not [46]. In FY 2021, those born in FY 2001, whose cumulative vaccination rate is 1.6%, will turn 20 years old, and they will be eligible for the first time for free cervical cancer screening. The year-over-year rate of cervical cytology abnormalities has been increasing steadily. The warnings sent to Japan by the GACVS in 2015 were not heeded, and Japan is slipping into a stage where the “real harm” will become a reality.

Vaccine hesitancy is caused by a complex decision-making process that is affected by a variety of contexts, such as individual, group, and vaccine-specific factors that include media, history, religion, culture, and socioeconomics [47]. In Ireland, a steering group of concerned organizations was established in 2016, and the HPV Vaccination Alliance was established in 2017 after the activities of anti-vaccine lobbying groups established in 2015 led to a decline in the Irish vaccination rate [48]. Forming powerful cross-sectional alliances led to a reversal and a rapid improvement in the Irish vaccination rate. The decline of HPV vaccination in Denmark has also been reversed through a national information campaign about the vaccine’s safety and effectiveness [49]. In order to revive the HPV vaccination rate in Japan, we have to first learn important lessons from the efforts of other countries and understand the unique factors in Japan involved in vaccination recommendations, including media, history, values, and culture. Then, the government, educational institutions, and medical institutions must work together to inform parents and children about the safety and efficacy of the HPV vaccine and respond to their concerns and questions.

In Japan, hesitancy to use the COVID-19 vaccines was reported to be higher among young women, and the most common reason for their hesitancy was fear of adverse events [50]. Other factors associated with their hesitancy were living alone and being of low socioeconomic status. We need to pick up on these findings and work to reintroduce the HPV vaccine. The Japanese governmental decisions were not made based on scientific evidence. They must devise a review of their decision system to ensure that this type of mistake will never be repeated [19,39,41,46]. In addition, the media in Japan should be mindful of their medical reporting with respect to scientific evidence [51]. We have repeatedly stressed the need to take the actions shown in Table 2 as soon as possible [52]. The Japanese government should be deeply aware of the health hazards caused by their decision-making.

## 7. Conclusions

It will be difficult for Japan to catch up with countries like Australia that have successfully introduced the HPV vaccine. However, we must return to the fight for the elimination of cervical cancer as soon as possible. The government must resume its recommendation and strongly promote effective cervical cancer control measures.

## Figures and Tables

**Table 1:** Timeline of the policies and events related to HPV vaccines in Japan.

2009	October	The bi-valent HPV vaccine was licensed.
2010	November	Subsidies from local and national governments for an HPV vaccination program for girls 13–16 commenced.
2011	July	The quadra-valent HPV vaccine was licensed.
2013	April	The national immunization program for girls aged 12–16 years commenced
	June	The VARRC ruled that “It is necessary to determine the frequency of pain occurrence whose relationship can be undeniably linked to HPV vaccination. HPV vaccination should not be actively recommended until proper information can be provided to the public.” The VARRC announced the suspension of its recommendation for vaccination (Notification by the Director-General of the Health Service Bureau of the MHLW).
2014	January and July	The VARRC evaluated the pathogenesis and causal relationship of the “diverse symptoms” reportedly experienced after HPV vaccination. The reported chronic pain and motor impairment were regarded as functional physical symptoms (a form of functional somatic syndrome).
2015	August	The “Guide for the Management and Treatment of Symptoms that Occur after HPV Vaccine Injection” was published. An organization of cooperative medical institutions from all 47 prefectures agreed to provide treatment for any girl suffering from symptoms after HPV vaccination in any community throughout Japan. The MHLW announced three measures for patients with symptoms, mainly of pain or movement disorders.
	September	The result of the adverse events follow-up survey was released. The suspension of governmental recommendation was continued. The MHLW and the Ministry of Education, Culture, Sports, Science, and Technology issued their “Improvement of the Consultation and Support System for Persons with Symptoms after HPV Vaccination”. Relief (subsidies for medical expenses, etc.) based on the Immunization Law and the Pharmaceuticals and Medical Devices Agency Law, was implemented
	November	Symptom consultation services were established in the health and education departments of each prefecture.
	December	The VARRC evaluated the safety and efficacy of HPV vaccines in Japan and abroad.
2017	April	The conclusions of a nationwide epidemiological survey by a research team designated by the MHLW were reported to the VARRC (Key finding: Unvaccinated girls had a similar number of “diverse symptoms”).
	November	The VARRC evaluated all available information on the safety and efficacy of the HPV vaccine in Japan and abroad and expressed its commitment to continue to provide close support to patients who presented with any of the diverse symptoms. The VARRC discussed ways to better inform the public about the HPV vaccine.
2018	January	An extensively revised informational leaflet was released by the MHLW to better inform the public about the HPV vaccine.
2019	August	The VARRC reported the results of a survey on the provision of HPV vaccine information.
2020	July	The “Parliamentary Association for the Resumption of Recommendation of HPV Vaccination” submitted a petition to the MHLW. The 9-valent HPV vaccine was licensed in Japan.
	October	MHLW’s ‘Leaflet to Inform the Public about the HPV Vaccine’ was revised.
	December	The quadra-valent HPV vaccine was approved to prevent anal cancer for males.
2021	August	“Parliamentary Association for the Resumption of Recommendation of HPV Vaccination” submitted a petition to the MHLW again. The Japanese Society of Obstetrics and Gynecology submitted a petition to the MHLW to extend the period of routine vaccination.

Abbereviations: human papillomavirus, HPV; Vaccine Adverse Reactions Review Committee, VARRC; Ministry of Health, Labor, and Welfare, MHLW.

**Table 2:** Issues which the MHLW should be considered.

1. Easy access to vaccination should be provided for women who are older than the normally targeted ages of 12–16 years who were not vaccinated during the vaccine recommendation hiatus.
2. The 9-valent vaccine should be introduced into a national immunization program.
3. Start a routine immunization program for boys of the same ages as the targeted girls.
4. Cervical cancer screenings should be especially strongly recommended for women who have not been vaccinated.
5. Special national action to revive HPV vaccination based on a behavioral economics approach.
6. Provide accurate vaccination information to the media.
7. Establish a vaccination registry and to verify the safety and efficacy of the vaccine.
